# Proceedings of New Orleans Medical and Surgical Association

**Published:** 1879-03

**Authors:** F. Loeber, Thos. Layton, I. L. Crawcour


### PROCEEDINGS OF NEW ORLEANS MEDICAL AND SURGICAL ASSOCIATION.

*Report of Special Committee on Yellow Fever and the best Measures for Preventing its Recurrence in New Orleans.*

In view of the efforts now being made to establish either a system of absolute non-intercourse between New Orleans and the West Indies, or a quarantine so restrictive in its laws as to virtually suspend all business, systems which have only existed during barbarous ages, which have been abolished by the good sense and practical knowledge of civilized nations, and which to use the words of John Simon, are but "paper plausibilities," we, the members of the Medical and Surgical Association of New Orleans, in order that our own people and the people of this country (at large) may not be misled by our silence, thus taking it as an endorsement of these schemes as proposed, deem it proper to come forward and enter our protest against such mersures and to suggest such means as we believe can alone render New Orleans a healthy city and free from epidemics of yellow fever.

We believe that yellow fever is a specific disease, depending upon specific cause, originally an exotic, now domesticated, and does not need a fresh importation either to produce sporadic cases or epidemics of yellow fever. This being our conviction, strengthened by the fact that quarantine *has never prevented* sporadic cases of yellow fever, nor ever will do it, we protest against the present system of quarantine or any one similar to it, or absolute non-intercourse, and can only endorse such a system of rational quarantine as shall cause least interference with our commercial relations and afford at the same time all the possible protection we can expect.

We believe that sanitary measures are of the most important, but are most neglected. After every epidemic committees have been appointed to investigate the causes of disease; their reports constantly speak of the terrible condition of the city in regard to sanitary matters. For the present year, the admirable report of Dr. Joseph Holt will serve as an example. All the records, from that of Dr. Fenner, in 1846, to those of the present year, lead to the almost certain conviction that the disease is epidemic, and not imported, depending on certain ill-understood climatic conditions and certain well understood defects in sanitation; but the result was and we fear will be always the same, no improvement in regard to hygiene, but stricter quarantine. The sanitary condition of New Orleans for years past has been, and even at the present day, is so defective, that we are surprised that not only yellow fever, but other pestilences, do not exist all the year round; but so great is the force of habit, that some of the inhabitants have declared that filth was healthy and should not be disturbed. What will the people of this country say when we show them that New Orleans, with a population of over 225,000, and an area of many square miles, has only a few paved streets; the rest are mere mud pathways, impassable to vehicles after a heavy rain, and composed largely of the filth and garbage of the city?

The drainage of the city, if it can be called by that name, is so bad, that after a heavy rain, most of the rear and some of our principal streets are overflowed. Many of the lots are lower than the streets, and water naturally accumulates in them, and stagnates. The privies of the whole city are so badly constructed that mechanics and sanitary officers have stated that none, after having been built for two years, will retain their contents, but are in constant communication with the ground water, so that we can safely say that the whole city is one vast cesspool, and has one privy in community. As drinking water we use that stored for months in cisterns, which are rarely if ever cleaned; the impurities therein contained need no microscope for detection, and when a drought occurs, which is not frequent, the masses have no water for drinking or ablusions, except what is obtained from the gutters, which in such seasons are filled by the fire plugs—these gutters probably not cleaned for weeks or months.

The law prescribes that the offal and garbage of the houses shall be placed in a barrel or box in front of each house, and shall be removed before 10 A. M. In many of the streets it remains for days and weeks, and when removed is simply carted a few blocks away, and deposited to fill a hole in the middle of the street, or a lot of ground in the heart of the city. This was especially conspicuous during the past summer, notwithstanding the repeated complaints of the Board of Health.

Our levees, as every one knows, are in the same miserable condition, filled with refuse and decomposing dejecta of all kinds, tainting the entire city.

We, therefore, recommend as absolutely necessary:

1. Proper drainage, underground sewerage, and the total abolition by law of the present system of privies.

2. A copious supply of pure water.

3. Paving of all the streets, and the filling up of all lots with river sand or gravel, these to be raised above the level of the street.

4. That instead of the present system of depositing the filth and offal collected by scavengers in the middle of the street, in empty lots, or in the rear of the city, the authorities shall have all such filth and offal thrown into the current of the river, below the city, by proper barges, such as are at present used by the vidangeurs of the city.

5. We especially recommend the taking of all proper measures with regard to the sanitary regulations of our graveyards, and such improvements in the system of burial of the destitute poor by the city as will prevent the nuisances and scenes which were described in the report presented by Dr. Holt to the city authorities and to the Board of Health in March, 1877.

F. Loeber, M.D.,

Thos. Layton, M.D ,

I. L. Crawcour, M.D.

